# Supplementary material for: Differential Metabolites and Metabolic Pathways Involved in Aerobic Exercise Improvement of Chronic Fatigue Symptoms in Adolescents Based on Gas Chromatography–Mass Spectrometry
Source: Int J Environ Res Public Health. 2022 Feb 18;19(4):2377. doi: 10.3390/ijerph19042377 (PMC8872503; doi:10.3390/ijerph19042377)
Supplement: Supplementary file 1 [file ijerph-19-02377-s001.zip › ijerph-1549121-supplementary.pdf]

Sample	Area	Sample	Area
QC 1	2976853	QC 6	3327254
QC 2	3580924	QC 7	3588170
QC 3	3791119	QC 8	4399838
QC 4	3422938	QC 9	4053487
QC 5	3545681	QC 10	4469056
**Average**	**3715532**	**RSD**	**12.69%**

Figure S1: Ion flow diagram of all samples.

It can be seen from the table that the relative standard deviation (RSD) of peak area of internal standard in QC sample is ≤30%, and the RSD is 12.69%, indicating that the instrument data collection is stable.

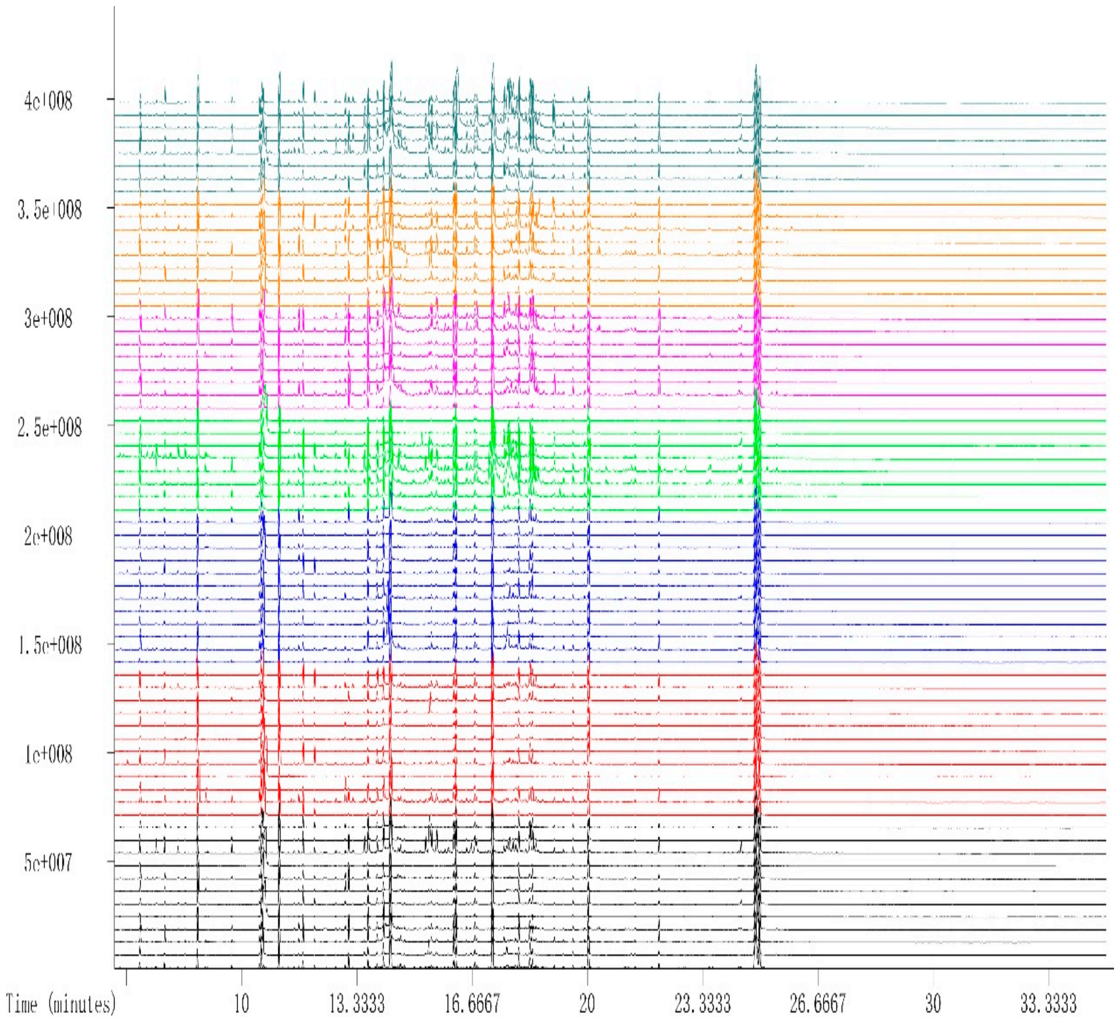

Figure S1 Ion flow diagram of all samples

Each line represents a sample
